# Variations in straw fodder quality and grain–Straw relationships in a mapping population of 287 diverse spring wheat lines

**DOI:** 10.1016/j.fcr.2019.107627

**Published:** 2019-11-01

**Authors:** Arun K. Joshi, Uttam Kumar, V.K. Mishra, Ramesh Chand, R. Chatrath, Rudra Naik, Suma Biradar, Ravi P. Singh, Neeraj Budhlakoti, Ravi Devulapalli, Michael Blümmel

**Affiliations:** a International Maize and Wheat Improvement Center (CIMMYT), NASC Complex, DPS Marg, New Delhi, India; b Borlaug Institute for South Asia (BISA), NASC Complex, DPS Marg, New Delhi, India; c Banaras Hindu University, Varanasi, India; d Indian Institute of Wheat and Barley Research (IIWBR), Karnal, India; e University of Agricultural Sciences, Dharwad, India; f International Maize and Wheat Improvement Center (CIMMYT), Apdo postal 6-641, Mexico DF, Mexico; g Centre for Agricultural Bioinformatics, Indian Agricultural Statistics Research Institute, Library Avenue, New Delhi, 110012, India; h International Livestock Research Institute (ILRI), ICRISAT Campus, Patancheru, Hyderabad 502324, Telangana, India; i International Livestock Research Institute (ILRI), P.O.Box5689, Addis Ababa, Ethiopia

**Keywords:** Wheat, Straw, Fodder traits, Mapping, GWAS

## Abstract

A wheat association mapping population consisting of 287 diverse spring wheat lines were evaluated for three years in one location (Varanasi) and out of these for one year across three locations (Karnal, Dharwad and Varanasi) in India. Straw fodder quality traits analyzed were nitrogen (N) content, neutral (NDF) and acid (ADF) detergent fiber, acid detergent lignin (ADL), ash (ASH), *in vitro* organic matter digestibility (IVOMD) and metabolizable energy (ME) content. Grain yield (GY) and straw yield (SY) were also recorded. Highly significant (P < 0.0001) differences among lines were observed for all traits except for ADF and ADL in the three years trials conducted at Varanasi. However, year and location had strong (P < 0.0001) effects on all traits. Compared to line-dependent variations in GY and SY variation in straw fodder quality traits were small. Proportionally greatest variations between lines were observed for straw N where lowest and highest N varied by about 30%. Difference for NDF and ADF between lines were at most 4% units and below 3% units for IVOMD. Grain yield and straw yield were positively correlated (P < 0.0001) with GY accounting for 26% of the variation in SY. Straw N, IVOMD and ME were weakly but significantly (P < 0.05) negatively associated with GY and SY. Straw NDF and ADF were significantly (P < 0.05) positively correlated with GY but the association was again weak. Straw NDF, ADF and ADL were also weakly but significantly positively correlated with SY.

Genome-wide association studies (GWAS) were applied to detect significant marker- straw fodder quality trait associations. Five genomic regions contributed for six traits (ADF, ADL, ASH, IVOMD, ME and NDF). ADF and ADL mapped in the common QTL region on chromosome 2B. Similarly, for the IVOMD and ME QTLs on chromosome 5B were associated with SNP marker, wsnp_Ku_c35090_44349517. While some associations were detected for ADF, ALD, ASH, IVOMD, ME and NDF on chromosomes 1A, 2B, 3A, 5A and 5B, the phenotypic variation explained was low to medium by individual QTL. A likely contributing factor was the comparatively small difference in straw fodder quality traits among the lines. It is interesting to note that line dependent variations in GY and SY were about two-fold. In other words, strong genotypic variations of GY and SY do exist. The lack of any similar variations in straw fodder quality traits is intriguing and requires further research.

## Highlights

• A wheat population of 287 diverse spring wheat lines were evaluated in a multilocation trial.
• Genome-wide association studies (GWAS) were applied to detect significant marker- straw fodder quality trait associations.
• Five genomic regions contributed for six traits (ADF, ADL, ASH, IVOMD, ME and NDF).
• ADF and ADL mapped in the common QTL region on chromosome 2B. Similarly, for the IVOMD and ME, QTLs were found on chromosome 5B.
• Strong genotypic variations of GY and SY do exist. The lack of any similar variations in straw fodder quality traits is intriguing and requires further research.

## 1 Introduction

In all countries of South Asia including India, wheat straw is a major resource of feed for the cattle rearing farmers and the dairies (Suresh et al., 2012; Blümmel et al., 2012). Wheat straw has always played an important role in agriculture and in rural societies of South Asia, where they are used for numerous purposes. Over the past several years, baling and selling wheat straw has become a more common practice. The increased demand of straw is driven by livestock farms using straw as part of their feed rations. The demand for wheat straw continues throughout the year but its availability depends on the wheat harvest season that in south Asia mostly occurs in the months of March-April (Teufel et al., 2010). The period March-May is the peak period for availability of wheat straw, therefore the period just before the harvest time, in the month of December to February, the cost of wheat straw peaks. At this peak price period, the straw-grain cost ratio varies between 0.30 to 0.48% compared to normal ratio of about 0.19 to 0.35 (Teufel et al., 2010). Since wheat straw is traded in huge quantity, the value of the straw is a very popular concern among farmers, especially small and marginal ones, who see wheat straw as an additional source of income. The price of straw is not uniform and may vary according to the grade and provenience of straws though this is more apparent in wholesale trading at village level where wheat cultivation is done. In urban markets varietal sources of straw are often not clearly defined (Teufel et al., 2010; Blümmel et al., 2012). In general, traders classify wheat straw as Best, Good, Medium and price differences between Best and Medium range from about 10% in urban to about 17% in village level trading (Teufel et al., 2010; Blümmel et al., 2012).

Wheat straw contains variable amount of nutrients which may vary depending upon cultivars (Kernan et al., 1979; Vaswani et al., 2013), nutrient management in the soil, environmental conditions during the growing season and the manner it is handled after the grain is harvested but to wheat improvement the genetic effect are particularly important. During last decade, Genome Wide Association Studies (GWAS) have been frequently used along with quantitative trait loci (QTL) mapping to understand the genetic bases of various traits in different crops (Crossa et al., 2014). GWAS have been used widely to analyze the genetic control of complex traits in wheat. However, to date, no QTL study is reported for wheat straw traits using conventional or GWAS approaches. The traditional QTL mapping approach often locates genomic regions containing polymorphisms that are limited to the biparental population and with low resolution. GWAS, as a complement to QTL mapping, has rapidly become a promising approach to genetic mapping based on linkage disequilibrium (LD). For high mapping resolution using GWAS, a large number of molecular markers are needed that can cover the whole genome at a sufficient resolution (Crossa et al., 2014). Because single nucleotide polymorphism (SNP) markers are abundant and evenly distributed across most genomes, they satisfy the large samples and high-density marker requirements of GWAS. Recently, the 90 K iSelect SNP genotyping array permitted a dramatic increase in the numbers of gene-based SNP markers that can be used to construct high-density linkage maps (Wang et al., 2014).

In this study an attempt was made to measure variation in 287 diverse genotypes based on testing in three different locations over two years in India. GWAS was also used to see whether there is possibility of identification of molecular markers associated with straw traits.

## 2 Materials and methods

### 2.1 Plant material used

The wheat association mapping initiative (WAMI) population consisting of 287 elite lines of spring bread wheat was obtained from CIMMYT`s Global Wheat Program at Mexico. The lines in this panel were of diverse pedigree but had low variation for flowering time and plant height to avoid confounding effects (Lopes et al., 2012). This population was earlier used to characterise lines for heat and drought stress tolerance traits and QTL identification for fine mapping and gene discovery (Lopes et al., 2012, 2015).

### 2.2 Phenotyping for straw and agronomic traits

A WAMI population was evaluated for three years in one location (Varanasi) and for one year across three locations in India (Karnal, Dharwad and Varanasi). The soils at Karnal, Dharwar and Varanasi locations were loamy, red and alluvium respectively. The trials were planted in the main spring wheat season in the recommended sowing time of November and harvested in the month of March to April depending on individual environments. Trials were grown under four irrigations with recommended fertilizer application of 120 Kg N: 80 Kg P: 60 Kg K per hectare. The trial was planted according to a randomized alpha lattice with two replications per line. The plots used were flat beds of 2.5–3.0 m long with 4 to 6 crop rows (20 to 23 cm between rows) with seed rate of 120 Kg/ha. The first irrigation was given at 21 days after planting. Remaining irrigations were given around a gap of three week. Each plot gave harvestable area of >3 m^2^. Grain yield (kg/ha) was determined using standard protocols (see Pask et al., 2012). Straw yield (kg/ha) was recorded by deducting grain yield from the total weight of the plot produce that included all biomass harvested from the base.

### 2.3 Laboratory fodder quality traits of wheat straw

Straw fodder quality traits analyzed were nitrogen (N) content, neutral (NDF) and acid (ADF) detergent fiber, acid detergent lignin (ADL), ash (ASH), *in vitro* organic matter digestibility (IVOMD) and metabolizable energy (ME) content. Grain (GY) and straw yield (SY) were also recorded. Straw samples were analyzed by Near Infrared Spectroscopy (NIRS), calibrated for this experiment against conventional wet laboratory analyses. The NIRS instrument used was a FOSS Forage Analyzer 5000 with software package WinISI II. Three hundred samples were selected based on spectra variations to develop NIRS equations for these traits and with these equations, the values of the remaining samples were blind predicted. For agreements between NIRS blind predicted values and conventionally determined values, see Joshi et al. (2019).

### 2.4 Genotyping and population structure

The population was genotyped following the methodology given in Lopes et al. (2015). The genotyping was done with the 9 K Infinium iSelect Beadchip SNP array. These markers were analyzed for polymorphism, minor allele frequency (>5%), and missing values. A detailed analysis of population stratification that influence the discovery of marker-trait associations was performed through a model-based clustering approach using the STRUCTURE software v. 3.0 (Pritchard et al., 2000) in which a Bayesian approach identifies clusters based on Hardy-Weinberg equilibrium and linkage disequilibrium.

### 2.5 Statistical analysis

The model: Yijk=μ+ti + bj+(tb)ij + eijk was used where *Y_ijk_* represents the *k*-th observation of *j*-th location or year (*j* = 1, 2, …a) on the *i-*th genotype (*i* = 1, 2, …, b). Therefore,  overall mean effect, ti represents the *i*-th genotype effect, bj represents jth location or year effect, (tb)ij is interaction effect on i-th genotype and *j*-th location or year and eijk represents the random error. The errors eijk are assumed to be normally and independently (NID) distributed, with mean zero and variance σ^2^_e_. SAS 9.4 (2012) statistical package was used for analysis of variance (ANOVA) by general linear model (PROC GLM), Comparison of means between treatments using Fisher’s least significance difference (LSD) test at 5% level of significance. Simple correlations among traits was used PROC CORR procedure. In Estimation of heritability (genotypic variance components / phonotypic variance components), the variance components of the traits were calculated by using the mixed model with restricted maximum likelihood (REML) method for evaluate heritability by PROC VARCOMP procedure.

Statistical analysis for GWAS were performed using SAS PROC MIX by adjusting for variability using the alpha lattice design. Least mean estimates were calculated for each trait. Phenotypic correlation of all the combination of traits was also estimated. A dataset including 287 lines was obtained after combining phenotypic and genotypic data. Genome-wide scans in TASSEL (Bradbury et al., 2007) using 5623 markers with known positions were conducted using population structure (Q2 to Q6) as the fixed component and a K matrix (kinship matrix) as the random component after model testing. Model comparison was done in SAS in the mixed model framework using the Bayesian information criteria (BIC) to select the best model for testing marker trait associations. Simple model, population structure as a cofactor (Q) (Pritchard et al., 2000), K matrix and random term in the mixed model, model involving population structure and familial relatedness (Q + K) (Yu et al., 2006), and principal components (PCs) from principal component analysis (PCA) and PCs + K were tested. A combination of Q + K model with model selection enabled was used to detect markers using Genome Association and Prediction Integrated Tool (GAPIT). GAPIT is an R based program that considerably reduces the computing time required for association mapping (R Development Core Team, 2010). Furthermore, results were verified in SAS by applying unified mixed model analysis (Yu et al., 2006). The threshold for defining a marker to be significant was taken at 10^3^ considering the small number of markers and the deviation of the observed F-test statistics from the expected F-test distribution.

## 3 Results

### 3.1 Variations in grain (GY) and straw (SY) yields and straw fodder quality traits and the effect of years

The means, ranges and statistical variations in grain (GY) and straw yields (SY), straw nitrogen (N), neutral (NDF) and acid detergent (ADF) fiber, acid detergent lignin (ADL), *in vitro* organic matter digestibilities (IVOMD) and metabolizable energy (ME) content in 287 spring wheat grown for 3 consecutive years in Varanasi in India are reported in Table 1. Except for ADF and ADL, highly significant (P < 0.0001) differences were observed for all traits. Lines varied by 2.1-fold for GY and by 1.9-fold for SY. In contrast, variations in straw quality traits were moderate being about 30% for straw and about 5% for NDF, IVOMD and ME.

**Table 1:** Means, ranges and statistical variations in grain (GY) and straw yields (SY), straw nitrogen (N), neutral (NDF) and acid detergent (ADF) fiber, acid detergent lignin (ADL), *in vitro* organic matter digestibilities (IVOMD) and metabolizable energy (ME) content in 287 spring wheat grown for 3 consecutive years in Varanasi in India.

Trait	Mean	Range	P < F
GY (kg/ha)	3677	2655–5464	<0.0001
SY (kg/ha)	4313	2888–5350	<0.0001
N (%)	0.81	0.68–0.98	<0.0001
NDF (%)	75.6	73.7–77.7	<0.0001
ADF (%)	50.9	49.5–52.4	0.21
ADL (%)	6.3	5.8–6.8	0.5
IVOMD (%)	49.9	48.4–51.2	<0.0001
ME (MJ/kg)	7.2	7.0–7.4	<0.0001

Year had a persistently greater effect on traits than line, and line by year effects were largely insignificant (Table 2 about here).

**Table 2:** Effects of line and year and their potential interactions on grain (GY) and straw yields (SY), straw nitrogen (N), neutral (NDF) and acid detergent (ADF) fiber, acid detergent lignin (ADL), *in vitro* organic matter digestibilities (IVOMD) and metabolizable energy (ME) content) in 287 spring wheat grown for 3 consecutive years in Varanasi in India.

	Grain Yield		Straw Yield		Straw Nitrogen		Straw NDF		Straw ADF		Straw ADL		Straw IVOMD		Straw ME	
Source	F-value	P > F	F-value	P > F	F-value	P > F	F-value	P > F	F-value	P > F	F-value	P > F	F-value	P > F	F-value	P > F
Line	1.4	0.0001	1.07	0.23	1.38	0.0003	0.62	0.9	0.99	0.55	0.73	0.9	0.85	0.9	0.84	0.9
Year	1378	<0.0001	5381	<0.0001	323	<0.0001	283	<0.0001	24.4	<0.0001	90	<0.0001	270	<0.0001	313	<0.0001
Line × Year	1.02	0.41	1.13	0.05	1.06	0.24	0.71	0.9	1	0.49	0.9	0.9	0.75	0.9	0.77	0.9

### 3.2 Variations in grain (GY) and straw (SY) yields and straw fodder quality traits and the effects of location

The means, ranges and statistical variations in GY, SY, straw N, NDF, ADF, ADL, IVOMD and ME in 267 out 287 spring wheat lines grown for one year at three locations in India (Karnal, Dharwad and Varanasi) are reported in Table 3. Highly significant (P < 0.0001) differences among lines were observed for all traits. As observed for when the mapping population was grown for three consecutive years in one-location variations among lines in GY and SY were far more substantial than variations in straw quality traits. Broad sense heritabilities ranged from h^2^ = 0.20 to h^2^ = 0.36 for straw quality traits but were quite low for GY (h^2^ = 0.14) and SY (h^2^ = 0.06).

**Table 3:** Means, ranges and statistical variations in grain (GY) and straw yields (SY), straw nitrogen (N), neutral (NDF) and acid detergent (ADF) fiber, acid detergent lignin (ADL), *in vitro* organic matter digestibilities (IVOMD) and metabolizable energy (ME) content in 287 spring wheat grown for one year at Karnal, Dharwad and Varanasi in India.

Trait	Mean	Range	P < F	h^2^
GY (kg/ha)	5101	3540–6718	<0.0001	0.14
SY (kg/ha)	8147	5457–12,328	<0.0001	0.06
N (%)	0.69	0.58–0.83	<0.0001	0.20
NDF (%)	76.4	74.2–78.1	<0.0001	0.32
ADF (%)	51.3	49.6–52.8	<0.0001	0.20
ADL (%)	6.1	5.8–6.4	<0.0001	0.36
IVOMD (%)	49.2	48.0–50.6	<0.0001	0.22
ME (MJ/kg)	7.1	7.0–7.3	<0.0001	0.20

While line had always a significant (P = 0.04) effect on traits the effect of location was stronger (P < 0.0001), though significant line x location interactions were only observed for GY and SY, (Table 4).

**Table 4:** Effects of line and location and their potential interactions on grain (GY) and straw yields (SY), straw nitrogen (N), neutral (NDF) and acid detergent (ADF) fiber, acid detergent lignin (ADL), *in vitro* organic matter digestibilities (IVOMD) and metabolizable energy (ME) content) in 287 spring wheat grown for one year at Karnal, Dharwad and Varanasi in in India.

	Grain Yield		Straw Yield		Straw Nitrogen		Straw NDF		Straw ADF		Straw ADL		Straw IVOMD		Straw ME	
Source	F-value	P > F	F-value	P > F	F-value	P > F	F-value	P > F	F-value	P > F	F-value	P > F	F-value	P > F	F-value	P > F
Line	1.8	<0.0001	1.85	<0.0001	1.19	0.04	1.34	0.001	1.2	0.03	1.5	<0.0001	1.26	0.008	1.24	0.01
Location	2999	<0.0001	1575	<0.0001	2680	<0.0001	1893	<0.0001	6890	<0.0001	2245	<0.0001	186	<0.0001	201	<0.0001
Line × Location	1.54	<0.0001	1.73	<0.0001	0.91	0.88	0.88	0.94	0.89	0.92	0.94	0.77	0.98	0.57	0.99	0.53

### 3.3 Relationships between in grain straw traits

There was positive correlation between GY and SY (P < 0.0001) with GY accounting for 26% of the variation in SY (Table 5). The positive straw quality traits N, IVOMD and ME were weakly (r = ―0.129 to ―0.24) though significantly (P < 0.05) negatively associated with GY in the both three years and the one-year trial. Only N was significantly negative correlated with SY in both trials. The fiber major constituents NDF and ADF both negative quality traits were significantly positively correlated with GY and SY in the one-year trials but not in the three years trials. However, all relationships, though statistically significant were weak.

**Table 5:** Correlations between grain (GY) and straw yields (SY) and straw nitrogen (N), neutral (NDF) and acid detergent (ADF) fiber, acid detergent lignin (ADL), *in vitro* organic matter digestibilities (IVOMD) and metabolizable energy (ME) content in 287 spring wheats.

Trait	SY (kg/ha)	N (%)	NDF (%)	ADF (%)	ADL (%)	IVOMD (%)	ME (MJ/Kg)
Three consecutive years in Varanasi							
GY (kg/ha)	0.07 (0.17)	−0.21 (0.0002)	0.06 (0.29)	−0.04 (0.51)	−0.08 (0.17)	−0.15 (0.008)	−0.12 (0.04)
SY (kg/ha)		−0.15 (0.009)	0.09 (0.1)	0.1 (0.09)	0.006 (0.92)	−0.06 (0.25)	−0.04 (0.47)
One year in Karnal, Dharwad and Varanasi							
GY (kg/ha)	0.51 (<0.0001)	−0.24 (0.0001)	0.14 (0.02)	0.25 (0.0001)	0.08 (0.17)	−0.15 (0.02)	−0.13 (0.04)
SY (kg/ha)		−0.13 (0.03)	0.14 (0.02)	0.23 (0.0001)	0.13 (0.04)	−0.11 (0.07)	−0.09 (0.12)

### 3.4 Genome-wide association studies (GWAS)

**Fig. 1:**
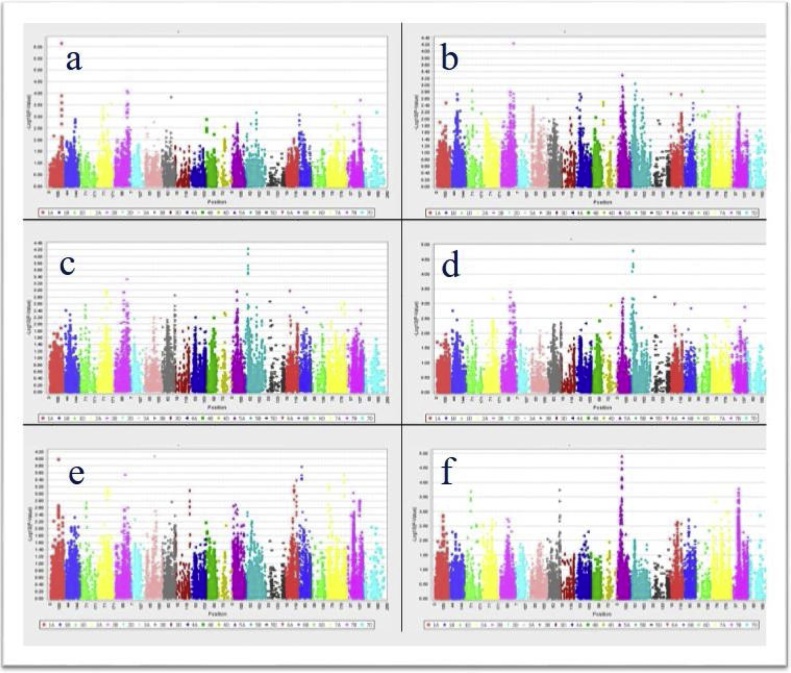
Manhattan plots showing significant QTLs across the genome associated with a) ADF b) ADL c) ASH d) IVOMD e) ME f) NDF.

The GWAS was applied to explore significant marker- straw fodder quality trait associations (Table 6). Some associations were observed for ADF on chromosomes 1A, 2B and for ADL on chromosome 2B. A significant association for IVOMD and ME was observed on chromosome 5B and for NDF and ASH on chromosomes 3A and 5A respectively (Fig. 1). The markers on chromosome 1A associated with ADF explained up to 11% of phenotypic variation. Although other associated markers were significant, however, the phenotypic variation explained was on the lower side (4.2–6.3%). A likely contributing factor were the comparatively small difference in straw fodder quality traits among the lines. It is interesting to note that line dependent variations in GY and SY were about two-fold. In other words, strong genotypic variations of GY and SY do exist.

**Table 6:** Significant markers associated with ADF, ADL, ASH, IVOMD, ME and NDF.

Trait	Marker	Chr	Pos	df	F value	P value	Marker R^2^	б^2^g	б^2^e
**ADF**	**wsnp_Ex_rep_c68058_66805898**	**1A**	**139**	**1**	**24.958**	**7.20E-07**	**0.1121**	**0.12694**	**1.05308**
ADF	Excalibur_c49875_479	2B	145	1	15.717	8.03E-05	0.0206	0.12694	1.05308
ADF	RAC875_c4602_445	2B	152	1	15.325	9.82E-05	0.098	0.12694	1.05308
**ADL**	**Excalibur_c49875_479**	**2B**	**145**	**1**	**16.337**	**5.83E-05**	**0.032**	**0.00264**	**0.02776**
**ASH**	**IACX7820**	**5A**	**56**	**1**	**19.341**	**1.24E-05**	**0.0916**	**0.05145**	**0.76203**
ASH	BS00100108_51	5A	56	1	18.419	2.00E-05	0.0874	0.05145	0.76203
ASH	BS00009531_51	5A	56	1	18.383	2.03E-05	0.0876	0.05145	0.76203
ASH	wsnp_Ex_c7383_12655468	5A	56	1	17.456	3.27E-05	0.083	0.05145	0.76203
ASH	wsnp_Ex_c7383_12654806	5A	56	1	17.375	3.42E-05	0.0855	0.05145	0.76203
ASH	wsnp_Ex_c7383_12655992	5A	56	1	16.086	6.65E-05	0.0791	0.05145	0.76203
**IVOMD**	**RAC875_c47084_378**	**5B**	**29**	**1**	**16.295**	**5.97E-05**	**0.0469**	**0.02517**	**0.56435**
IVOMD	wsnp_Ku_c35090_44349517	5B	29	1	15.615	8.50E-05	0.0398	0.02517	0.56435
**ME**	**wsnp_Ku_c35090_44349517**	**5B**	**29**	**1**	**18.762**	**1.68E-05**	**0.0633**	**3.59E-04**	**0.00925**
ME	BS00033612_51	5B	29	1	16.862	4.47E-05	0.0496	3.59E-04	0.00925
ME	Kukri_c74960_427	5B	29	1	16.335	5.85E-05	0.0434	3.59E-04	0.00925
**NDF**	**Tdurum_contig76105_124**	**3A**	**105**	**1**	**15.626**	**8.41E-05**	**0.0421**	**0.17025**	**1.64099**
NDF	Tdurum_contig76105_201	3A	105	1	15.566	8.68E-05	0.042	0.17025	1.64099

## 4 Discussion

### 4.1 Variations in wheat straw fodder quality and grain - straw relationships

Wheat straw is an essential feed resource in South Asia (Suresh et al., 2012) which can be improved by higher straw quantity and quality. In the present work straw quantity varied substantially among the lines (Table 1, Table 3) and selection and promotion of appropriate lines could certainly contribute to increased feed resources from wheat straw. However, the variations in straw quality traits, perhaps with the exception of straw N content, were comparatively small. For example, in multidimensional maize breeding exploring potential candidate genomic regions with implications for maize stover fodder quality in a panel of 276 inbred lines Vinayan et al. (2013) observed a difference in IVOMd between the lines of about 13 percentage points (47.9 to 60.6%). In the present work, differences among the lines in IVOMD were below 3 percentage points (Table 1, Table 3). These findings agree with observations by Joshi et al. (2019) and Blümmel et al. (2019a) from comprehensive overviews of wheat straw breeding work in South Asia who reported very limited genetic variation in wheat straw fodder quality.

Aforementioned observations however disagree with reports from older wheats varieties. For example, Kernan et al. (1979) investigated in multi-location trials six wheat cultivars over four years and IVOMD varied cultivar-dependent by 5.3 percentage points. In a more recent trial with five older wheat cultivars still popular in Northern India, Vaswani et al. (2013) observed cultivar-dependent differences in IVOMD of 7.7 percentage units. Such cultivar dependent variations in straw quality are in fact more in tune with work reported for a wide range of key cereal and legume crops (Blümmel et al., 2019b). In fact, one incentive in exploring the current mapping population was the expectation of finding higher genetic variability in straw fodder quality. The mapping population that was developed using lines having similar height and maturity to reduce confounding phenology. In earlier studies, this population showed significant genetic diversity for association mapping studies for agronomic and physiological traits (Lopes et al., 2012, 2015). The mapping population still showed very significant variations among lines in GY and SY. The lack of any similar variations in straw fodder quality traits is intriguing and requires further research.

### 4.2 Genome-wide association studies (GWAS)

The GWAS study lead to identification of molecular markers significantly associated with ADF, ADL, ASH, IVOMD, ME and NDF value on five chromosomes spread over A and B genomes (1A, 2B, 3A, 5A & 5B). However, the markers explained low to medium phenotypic variation (Table 6). This is promising, considering the comparatively small difference in straw fodder quality traits among the lines. It was interesting to note that the QTL for ADF and ADL shared the common QTL region. It is probably due to association between ADF and lignin content in the fodder (Holtzapple, 2003). Similarly, the QTLs for IVOMD and ME were linked to the common SNP marker (wsnp_Ku_c35090_44349517) on chromosome 5B. Although, phenotypic correlation between IVOMD and ME was not strong but still significantly positively correlated (P < 0.05). Earlier studies conducted on agronomic traits showed QTL for grain yield on chromosome 5B (Huang et al., 2003; Edae et al., 2014) and for other traits on different chromosomes (Pinto et al., 2010). Till date, no study has been done for mapping of straw nutrient traits in wheat. This study could not lead to any significant marker although some indications were found suggesting that in populations with significant variation for straw traits possibility for mapping can be explored. Once trait associations are obtained, they will be valuable as indirect selection criteria. Probably marker discovery for straw trait may focus on minor genes that show high G x E interaction. Wheat has a large genome size (17GB), is allohexaploidy with three different genomes (A, B, and D), and the unfinished genome sequence makes wheat a tough crop for marker discovery (Sukumaran and Yu, 2014).
